# Pilocarpine 1% for Improved Near Vision in Pseudophakic Patients

**DOI:** 10.18502/jovr.v20.15331

**Published:** 2025-05-19

**Authors:** Alireza Peyman, Maryam Naderi-Lordejani, Matin Irajpour, Mohammad Javad Ghanbarnia, Nima Koosha, Mohsen Pourazizi

**Affiliations:** ^1^Isfahan Eye Research Center, Department of Ophthalmology, Isfahan University of Medical Sciences, Isfahan, Iran; ^2^Department of Ophthalmology, Babol University of Medical Sciences, Babol, Iran

**Keywords:** Cataract Surgery, Pilocarpine, Presbyopia, Pseudophakia

## Abstract

**Purpose:**

To evaluate the efficacy and safety of 1% pilocarpine to enhance near vision in pseudophakic patients with monofocal intraocular lens (IOL).

**Methods:**

This prospective, nonrandomized, open-label, pre–post interventional study was conducted on patients who had a history of phacoemulsification and monofocal IOL implantation without any complications at least six months before the intervention. One drop of 1% pilocarpine solution was instilled in one of the patients' eyes, and near and distance visual acuity was assessed before and 20 minutes after monocular administration of the drop. Adverse effects were monitored for 8 hours.

**Results:**

We studied54 pseudophakic eyes of 54 patients with a mean age of 55.02 ± 10.10 years, including 36 males (66.7%). Distance-corrected near visual acuity improved significantly after administering 1% pilocarpine (*P* < 0.001). After treatment, no significant decrease in corrected distance visual acuity (CDVA) was observed (*P *= 0.60). Overall, 46 patients (85.2%) exhibited an improvement of more than one line in near vision after treatment; however, eight patients (14.8%) did not show any changes in near vision. Only two patients (3.7%) gained three lines in near vision. Headache, irritation, and nausea were observed in three patients, two patients, and one patient, respectively.

**Conclusion:**

The monocular 1% pilocarpine solution significantly improved near visual acuity among patients with pseudophakia and featured an acceptable safety profile. Although this improvement is considerable, it may not be sufficient for complete spectacle independence.

**Trial registration:** ClinicalTrials.gov identifier: NCT05578001

##  INTRODUCTION 

Presbyopia is characterized as a progressive age-related loss of near visual acuity due to diminished accommodation, which usually manifests after about 40 years of age. If left untreated, loss of near vision can severely impact one's quality of life.^[1]^ In a study conducted in 2015, it was estimated that 1.8 billion people, about 25% of the global population at that time, were affected by this disorder. The same study estimated that by 2030, this figure will increase to 2.1 billion people.^[2]^ Patients who undergo phacoemulsification lose their ability to focus on nearby objects; therefore, all pseudophakic patients are presbyopic.^[3]^ Considering the increasing number of cataract surgeries, most of which still involve using monofocal intraocular lenses (IOLs), there is an urgent need to conduct further studies in this field.^[4,5]^

Spectacles are generally the typical method of correcting presbyopia, but contact lenses are also widely used. To achieve spectacle independence, researchers have proposed a wide range of surgical techniques, such as presbyopic LASIK,^[6]^ corneal inlays,^[7]^ multifocal IOLs,^[8]^ and scleral implants.^[9]^ To achieve the same goal without any invasive surgeries, some authors have proposed pharmacological treatments such as miotic agents and lens softeners.^[10]^ The aforementioned pharmacological studies have focused explicitly on phakic patients.

A noteworthy step in this area was the approval of 1.25% pilocarpine for this purpose by the US Food and Drug Administration (FDA) in November 2021.^[11]^ Pilocarpine is a cholinergic muscarinic receptor agonist. Previous studies have indicated that pilocarpine can improve accommodation and depth of focus.^[12]^ However, in pseudophakic patients with non-accommodative IOLs, only the latter mechanism is operative. In fact, by decreasing the pupil size and subsequently increasing the depth of focus, this medication can improve near visual acuity in these individuals. The present study aimed to assess the efficacy and safety profile of pilocarpine 1% in improving near vision in monofocal pseudophakic patients.

##  METHODS

### Study Design 

This was a prospective, consecutive, non-randomized, open-label, pre–post interventional study conducted from April 2022 to September 2022 in Feiz Ophthalmology Referral Hospital, Isfahan University of Medical Sciences, Isfahan, Iran. It abided by the Helsinki Declaration of Ethical Principles and was approved by the Institutional Review Board of Isfahan University of Medical Sciences (IR.MUI.MED.REC.1401.184). The trial was also registered at the ClinicalTrials.gov (NCT05578001).

All participants provided written informed consent, and the original protocol was not changed after the trial's initiation. This trial report follows the Consolidated Standards of Reporting Trials (CONSORT) 2010 Statement and the CONSORT-Outcomes 2022 extension.

### Study Participants 

The recruited patients were over 40 years of age, with subjective and objective evidence of presbyopia. They had undergone phacoemulsification in both eyes using EnVista monofocal IOL (MX60, Bausch + Lomb, Bridgewater, NJ, USA) at least six months prior to the study and reported no complications. The patients had a best-corrected visual acuity (BCVA) of 20/25 or better in distance vision, refractive error of +1.50 to –0.50 diopters (D), and a cylinder of < 1.50 D.^[13]^ Pseudophakic individuals with non-accommodative IOLs were considered presbyopic due to lack of accommodation. The diagnostic criterion for presbyopia was defined as the need for a spectacle lens ≥ +1.00 D to read the print size of Jaeger 0.8.^[12]^ The exclusion criteria were as follows: lack of consent, high axial length ( > 26 mm), history of sensitivity to pilocarpine, use of any medications that interact with pilocarpine, use of any topical medications that can alter pupil size, and history of other ophthalmologic diseases such as amblyopia, glaucoma, and retinal or macular diseases.

### Study Intervention 

Before the intervention, patients' pupil size was measured in photopic conditions using a slit-lamp biomicroscope with maximum brightness to match the perfectly focused image of the iris with the length of the vertical light beam. Then, the number indicated in the slit-length display window was recorded as the pupil size, and the patients' iris color was documented based on the classification scale proposed by Simionescu et al.^[14]^ Then, the patients' uncorrected distance visual acuity (UCDVA) was measured using a Snellen chart at 6 meters. Next, according to the patients' refractive error, an appropriate spectacle was prepared to evaluate best corrected distance visual acuity (BCDVA). Finally, with the same spectacle, the patients' distance corrected near vision acuity (DCNVA) was measured and recorded using a Rosenbaum near vision card at 40 cm of distance. Then, one drop of pilocarpine 1% (Glaupin, Sina Daru Inc.) was administered only in the nondominant eye. After 20 minutes, the VA measurements were repeated. The dominant eye was determined using the Miles eye dominance test.^[15]^ The gained lines in the Jaeger chart were converted to logMAR for a more robust statistical analysis. Adverse effects such as headache, blurred vision, brow ache, eye pain, irritation, and nausea were recorded at 30 minutes and 8 hours after pilocarpine administration. Eyedrop administration was performed between 1 PM and 4 PM so patients could experience and report any potential nighttime symptoms.

### Outcome Measures 

The outcome measure for efficacy was defined as the number of monocular near visual acuity lines gained at 40 cm, using a Rosenbaum near vision chart, 20 minutes after drug instillation. The outcome measure for safety was defined as the number of patients without decreased CDVA at 6 meters, according to the Snellen chart.

### Statistical Analysis

All statistical analyses were conducted using SPSS version 26 (IBM Corp). Normal distribution of the data was evaluated using the Shapiro-Wilk test for normality. Continuous variables were presented using Mean ± SD or median and interquartile range (25 ${}^{\text{th}}$ quartile, 75 ${}^{\text{th}}$ quartile). Categorical variables are expressed as the number of cases (*n*) and percentage (%). The differences between the medians of pre- and post-treatment DCNVA and pre- and post-treatment CDVA were analyzed using the non-parametric Wilcoxon signed-rank test. Independent sample *t*-test and chi-square test were used to evaluate the difference in baseline characteristics between those who gained ≥ 2 near visual acuity lines and those who gained < 2 lines. Two-sided *P*-values < 0.05 were considered statistically significant.

##  RESULTS 

**Figure 1:**
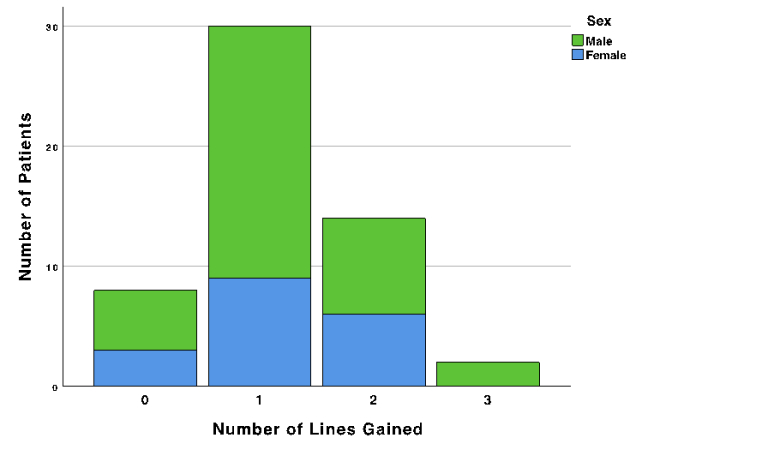
Near vision lines gained, stratified by sex.

Overall, 54 eyes of 54 patients (mean age, 55.02 ± 10.10 years) were included in the final analysis. Baseline demographics and clinical characteristics of the patients are presented in Table 1. Most patients (66.7%) were male, and 29 (53.7%) were younger than 55 years of age. The results of DCNVA and CDVA before and after treatment with pilocarpine are presented in Table 2. Near vision improved significantly after treatment (median 0.70 [0.54-0.7] vs. median 0.4 [0.30-0.70]; *P* < 0.001). No significant decrease in CDVA was observed after treatment with pilocarpine (median 0.00 [0.00-0.04] vs. median 0.00 [0.00-0.04]; *P *= 0.60). Table 2 exhibits a significant improvement in near visual acuity regardless of patients' baseline characteristics. On average, near visual acuity improved by 1.19 ± 0.73 lines. The number of near visual acuity lines gained after treatment is demonstrated in Table 3. Overall, 46 patients (85.2%) achieved at least one line after treatment with pilocarpine, and 8 patients (14.8%) showed no improvement. No loss of line was observed in any of the patients. The number of near visual acuity lines gained in male and female patients is demonstrated in Figure 1.

Near vision improved by two or more lines in 16 patients (29.6%), while only 2 (3.7%) gained three lines. Table 4 compares the baseline characteristics between those who gained ≥ 2 lines following treatment and those who did not achieve this level of improvement. No significant difference was identified between the two groups regarding age, sex, iris color, or photopic pupil size (*P* > 0.05).

No major short-term adverse events were observed in this study. Eight patients (14.8%) experienced at least one minor adverse effect following the treatment. Nausea, headache, and irritation were observed in one (1.9%), three (5.6%), and two (3.7%) patients, respectively. Two patients (3.7%) experienced both headaches and reduced night vision.

**Table 1:** Baseline characteristics and demographics of participants

**Parameter**	**Participants (** ***n*** ** = 54)**
Age (yrs)	
Mean (SD)	55.02 (10.10)
Median [min, max]	52.50 ${}^{\left[\text{40}\text{to}\text{76}\right]}$
< 55	29 (53.7%)
≥ 55	25 (46.3%)
Sex	
Male, *n* (%)	36 (66.7%)
Female, *n* (%)	18 (33.3%)
Laterality	
OD, *n* (%)	30 (55.6%)
OS, *n* (%)	24 (44.4%)
Photopic pupil diameter, mm	
Mean (SD)	2.16 (0.49)
1.0 mm, *n* (%)	1 (1.9%)
1.5 mm, *n* (%)	6 (11.1%)
2.0 mm, *n* (%)	33 (61.1%)
2.5 mm, *n* (%)	3 (5.6%)
3.0 mm, *n* (%)	11 (20.4%)
Iris color	
Light brown, *n* (%)	14 (25.9%)
Dark brown, *n* (%)	40 (74.1%)
UCDVA, LogMAR, Median (IQR)	0.05 (0.00, 0.10)
CDVA, LogMAR, Median (IQR)	0.00 (0.00, 0.05)
DCNVA	
LogMAR, Median (IQR)	0.70 (0.54, 0.70)
≥ 0.7 (LogMAR), *n* (%)	32 (59.2%)
Spherical equivalent, Mean (SD)	
Mean (SD)	–0.41 (0.34)
Median [min, max]	–0.37 [–1.00, 0.38]
IQR, interquartile range; UCDVA, uncorrected distance visual acuity; CDVA, corrected distance visual acuity; DCNVA, distance corrected near visual acuity	

**Table 2:** Comparison of VA (LogMAR) before and after treatment, stratified by baseline characteristics

	***N*** ** (%)**	**DCNVA (LogMAR)**		***P*** **-value**	**CDVA (LogMAR)**		***P*** **-value**
		**Before treatment, Mdn (IQR)**	**After treatment, Mdn (IQR)**		**Before treatment, Mdn (IQR)**	**After treatment, Mdn (IQR)**	
All participants	54 (100.0 %)	0.70 (0.54-0.7)	0.4 (0.30,0.70)	< 0.001	0.00 (0.00-0.04)	0.00 (0.00-0.04)	0.604
Age group (yrs)							
< 55	29 (53.7%)	0.70 (0.40-0.70)	0.40 (0.30-0.54)	< 0.001	0.00 (0.00-0.02)	0.00 (0.00-0.02)	0.891
≥ 55	25 (46.3%)	0.70 (0.54-1.00)	0.54 (0.40-0.70)	< 0.001	0.04 (0.00-0.10)	0.00 (0.00-0.01)	0.496
Sex							
Male	36 (66.7%)	0.70 (0.54-1.00)	0.40 (0.32-0.70)	< 0.001	0.00 (0.00-0.4)	0.00 (0.00-0.04)	0.604
Female	18 (33.3%)	0.70 (0.40-0.70)	0.40 (0.30-0.58)	0.001	0.00 (0.00-0.06)	0.00 (0.00-0.06)	1.000
Iris color							
Dark brown	40 (74.1%)	0.70 (0.54-0.70)	0.40 (0.30-0.54)	< 0.001	0.00 (0.00-0.04)	0.00 (0.00-0.04)	0.604
Light brown	14 (25.9%)	0.70 (0.40-1.00)	0.54 (0.37-0.70)	0.005	0.02 (0.00-0.04)	0.02 (0.00-0.04)	1.000
Mdn, median; IQR, Interquartile range; CDVA, corrected distance visual acuity; DCNVA, distance corrected near visual acuity							

**Table 3:** Breakdown of the number of DCNVA lines gained after treatment, stratified by age, sex, iris color, and photopic pupil size

		**Number of lines gained after treatment, ** ***n*** (%)*				**Mean (SD)**
	***N*** ** (%)**	**0**	**1**	**2**	**3**	
All participants	54 (100.0)	8 (14.8%)	30 (55.6%)	14 (25.9%)	2 (3.7%)	1.19 (0.73)
Age group (yrs)						
< 55	29 (53.7%)	5 (62.5%)	15 (50.0%)	7 (50.0%)	2 (100.0%)	1.21 (0.82)
≥ 55	25 (46.3%)	3 (37.5%)	15 (50.0%)	7 (50.0%)	0 (0.0%)	1.16 (0.62)
Sex						
Male	36 (66.7%)	5 (62.5%)	21 (70.0%)	8 (57.1%)	2 (100.0%)	1.19 (0.75)
Female	18 (33.3%)	3 (37.5%)	9 (30.0%)	6 (42.9%)	0 (0.0%)	1.17 (0.71)
Iris color						
Dark brown	40 (74.1%)	4 (50.0%)	24 (80%)	12 (85.7%)	0 (0.0%)	1.20 (0.61)
Light brown	14 (25.9%)	4 (50.0%)	6 (20%)	2 (14.3%)	2 (100.0%)	1.14 (1.03)
Photopic pupil size (mm)						
1.0	1 (1.9%)	0 (0.0)	0 (0.0)	1 (7.1%)	0 (0.0%)	2.00 (0.00)
1.5	6 (11.1%)	1 (12.5%)	4 (13.3%)	1 (7.1%)	0 (0.0%)	1.00 (0.63)
2.0	33 (61.1%)	5 (62.5%)	19 (63.3%)	8 (57.1%)	1 (50.0%)	1.15 (0.71)
2.5	3 (5.6%)	1 (12.5%)	2 (6.7%)	0 (0.0%)	0 (0.0%)	0.67 (0.58)
3.0	11 (20.4%)	1 (12.5%)	5(16.7%)	4 (28.6%)	1 (50.0%)	1.45 (0.82)
${}^{*}$ Percentage within the number of lines gained DCNVA, distance corrected near visual acuity						

**Table 4:** Comparison of baseline characteristics between patients whose DCNVA improved by ≥ 2 lines and those with < 2 lines after treatment with pilocarpine

	**Number of NVA lines gained after treatment **		***P*** **-value**
	** < 2 ** ***n*** ** = 38**	** ≥ 2 ** ***n*** ** = 16**	
Age, yr, Mean (SD)	53.92 (10.23)	57.63 (9.56)	0.221
Sex			0.673
Male, *n* (%)	26 (68.4%)	10 (62.5%)	
Female, *n* (%)	12 (31.6%)	6 (37.5%)	
Iris color			0.920
Light brown, *n* (%)	28 (73.7%)	12 (75.0%)	
Dark brown, *n* (%)	10 (26.3%)	4 (25.0%)	
Photopic pupil size, mm, Mean (SD)	2.13 (0.44)	2.22 (0.60)	0.558
DCNVA, distance corrected near visual acuity			

**Table 5:** Summary of latest publications on using pilocarpine to treat presbyopia

***Study ***	***Formulation***	***Dosing***	***Study population***	***Assessment tool/ efficacy endpoint***	***Outcome ***
*Shafer* ^[22]^	Pilocarpine hydrochloride 1.25% (Pilo 1.25%; Vuity; Allergan, an AbbVie Company)	Twice daily	62 phakic adults aged 40–55 years; 28 in Pilo group and 34 in vehicle group	Presbyopia Patient Satisfaction Questionnaire (PPSQ)	75% of interviewed Pilo 1.25% users found it effective. None of the participants in the vehicle group reported a significant improvement.
*Holland* ^[23]^	CSF-1 (0.4% pilocarpine HCl)	Twice daily	613 phakic adults aged 45–64 years; 309 in Pilo group and 304 in vehicle group	Achievement of ≥ 3-line (15 letters) gain from baseline	40.1% of the Pilo group and 19.1% of the vehicle group achieved efficacy endpoint (*P* < 0.0001).
*Lievens* ^[24]^	Pilocarpine HCl 1.25% (Pilo; Vuity; AGN-190584; Allergan, an AbbVie Company)	Once daily	80 phakic adults aged 40–55 years with history of LASIK or PRK; 39 in the Pilo group and 41 in vehicle group	≥ 3-line improvement in mesopic, high-contrast, binocular DCNVA on day 30	Responder rates in the LASIK/PRK subgroup were significantly higher with Pilo than vehicle.
*Waring* ^[11]^	Pilocarpine HCl 1.25% (Pilo; Vuity; AGN-190584; Allergan, an AbbVie Company)	Once daily	323 phakic adults aged 40–55 years; 163 in Pilo group and 160 in vehicle group	≥ 3-line improvement in mesopic, high-contrast, binocular DCNVA on day 30	Pilo was found effective in improving DCNVA at 3 and 6 hours but not at 8 hours after administration.
*LASIK*,* laser*-*assisted in situ keratomileusis*;* PRK*, p*hotorefractive keratectomy*;* DCNVA*,* distance-corrected near visual acuity*					

##  DISCUSSION

In this study, 1% pilocarpine improved near visual acuity in most patients with pseudophakia. However, despite better near vision after treatment, only two patients achieved three lines of improvement. This level of improvement is not usually sufficient for total spectacle independence, which is the primary goal of pharmacologic treatments for patients with presbyopia.

Regarding the safety of this treatment, none of the patients experienced decreased distance visual acuity, and no significant adverse effects were observed. One primary concern in the long-term use of miotics, such as pilocarpine, is the risk of retinal detachment. Studies have not identified a direct cause-and-effect relationship between the use of miotics and retinal detachment.^[16]^ However, there is a case series of three eyes from two patients with retinal detachment following the use of pilocarpine for presbyopia.^[17]^ Given that patients with pseudophakia have a higher risk of retinal detachment, disclosing this potential adverse effect is of utmost importance.^[18]^

Despite the lack of evidence for long-term efficacy, 1.25% pilocarpine was approved by the US FDA for phakic presbyopia.^[10,11]^ Other drugs have still not been approved by the FDA or any major regulatory body for treating presbyopia. One crucial disadvantage associated with miotics is the dimness caused by insufficient light reaching the retina.^[19]^ This can be especially dangerous in low-light settings, and patients using miotics are warned about this issue.^[10,20,21]^

Table 5 summarizes the latest trials and their findings on using different formulations of pilocarpine to treat presbyopia. In phakic individuals, pilocarpine can improve both accommodation and depth of focus.^[11,21]^ However, since it can only improve the depth of focus in pseudophakia, it is expected to have decreased efficacy for treating presbyopia in pseudophakic patients.

In this study, pilocarpine was instilled only in the nondominant eye to assess the improvement in monocular near visual acuity. Despite numerous studies in this field, there is no established standard for either bilateral or unilateral administration of this medication.^[10]^ Each method has advantages and disadvantages. In the case of monocular application, dimness seems less of an issue, but it requires some degree of familiarity of the patient with monovision. Binocular application has better near-vision outcomes, but dimness is more pronounced in this method.^[19]^ Meanwhile, no study has directly compared these two treatment options.^[10]^

The most important limitation of the present study was the absence of a placebo group, which would have allowed the comparison of the results against those of the treatment group. Another limitation was the lack of a long-term follow-up. Future studies should compare the effects of pilocarpine on pseudophakic individuals versus phakic patients. Moreover, future studies should compare the efficacy and safety of topical miotics in monocular and binocular treatment methods.

In summary, monocular use of 1% pilocarpine can partly improve near vision in patients with pseudophakia, but this improvement is often not enough to warrant complete spectacle independence. Nevertheless, this method did not cause blurred distance vision. Therefore, it is a safe and low-cost treatment for presbyopia that can be administered in both pseudophakic and phakic patients. Also, in the monocular approach, the untreated eye prevents dimness and lowered brightness. Possibly, other miotics or higher concentrations of pilocarpine can have more prominent improvements.

##  Financial Support and Sponsorship

None.

##  Conflicts of Interest

None.
